# Supplementary material for: Transcriptome Analysis of the Fruit of Two Strawberry Cultivars “Sunnyberry” and “Kingsberry” That Show Different Susceptibility to Botrytis cinerea after Harvest
Source: Int J Mol Sci. 2021 Feb 3;22(4):1518. doi: 10.3390/ijms22041518 (PMC7913547; doi:10.3390/ijms22041518)
Supplement: Supplementary file 1 [file ijms-22-01518-s001.zip › Supplementary Figure.pptx]

## Slide 1
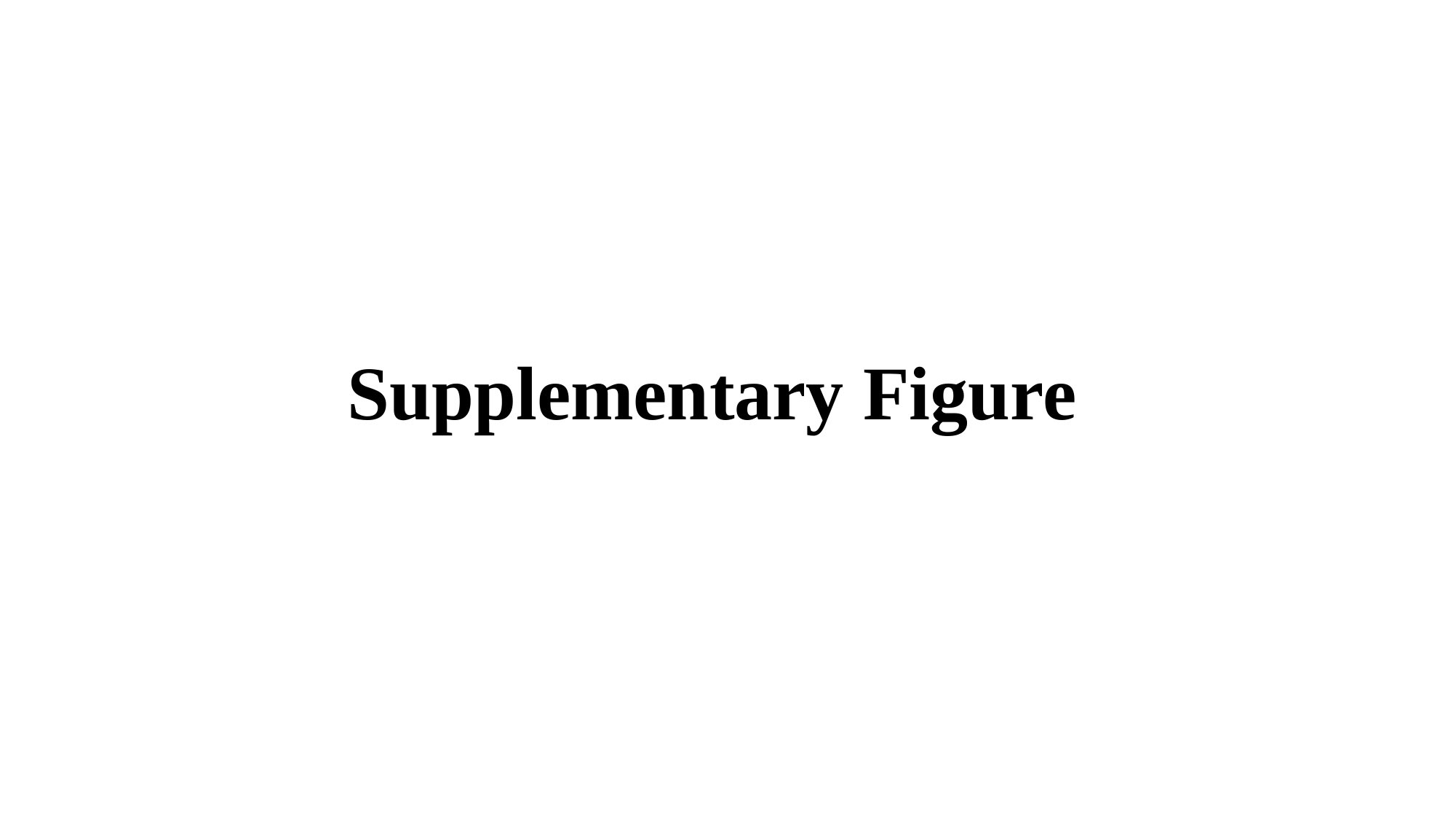

# Supplementary Figure

## Slide 2
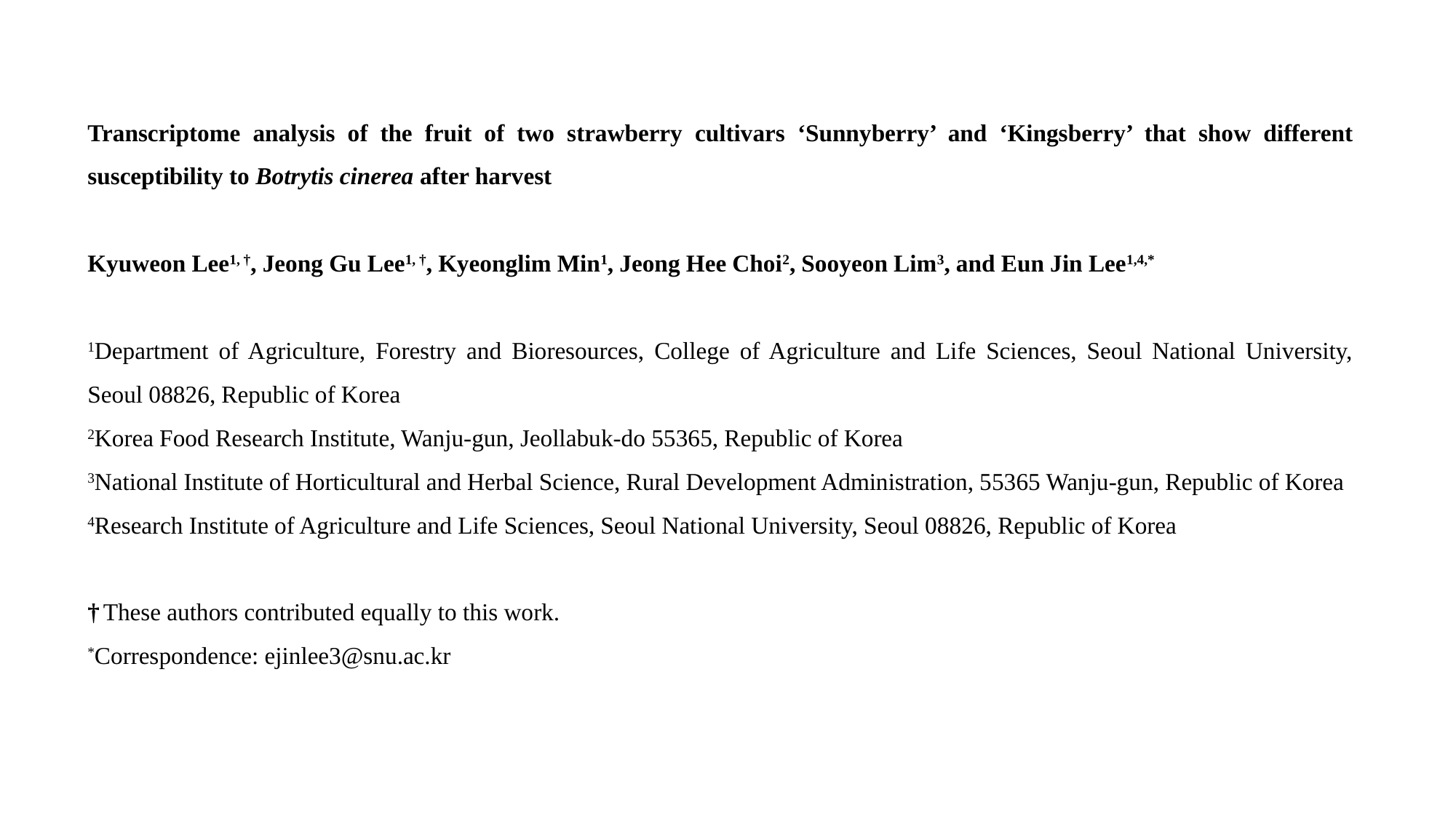

Transcriptome analysis of the fruit of two strawberry cultivars ‘Sunnyberry’ and ‘Kingsberry’ that show different susceptibility to Botrytis cinerea after harvest
Kyuweon Lee1, †, Jeong Gu Lee1, †, Kyeonglim Min1, Jeong Hee Choi2, Sooyeon Lim3, and Eun Jin Lee1,4,*
1Department of Agriculture, Forestry and Bioresources, College of Agriculture and Life Sciences, Seoul National University, Seoul 08826, Republic of Korea
2Korea Food Research Institute, Wanju-gun, Jeollabuk-do 55365, Republic of Korea
3National Institute of Horticultural and Herbal Science, Rural Development Administration, 55365 Wanju-gun, Republic of Korea
4Research Institute of Agriculture and Life Sciences, Seoul National University, Seoul 08826, Republic of Korea
† These authors contributed equally to this work.
*Correspondence: ejinlee3@snu.ac.kr

## Slide 3
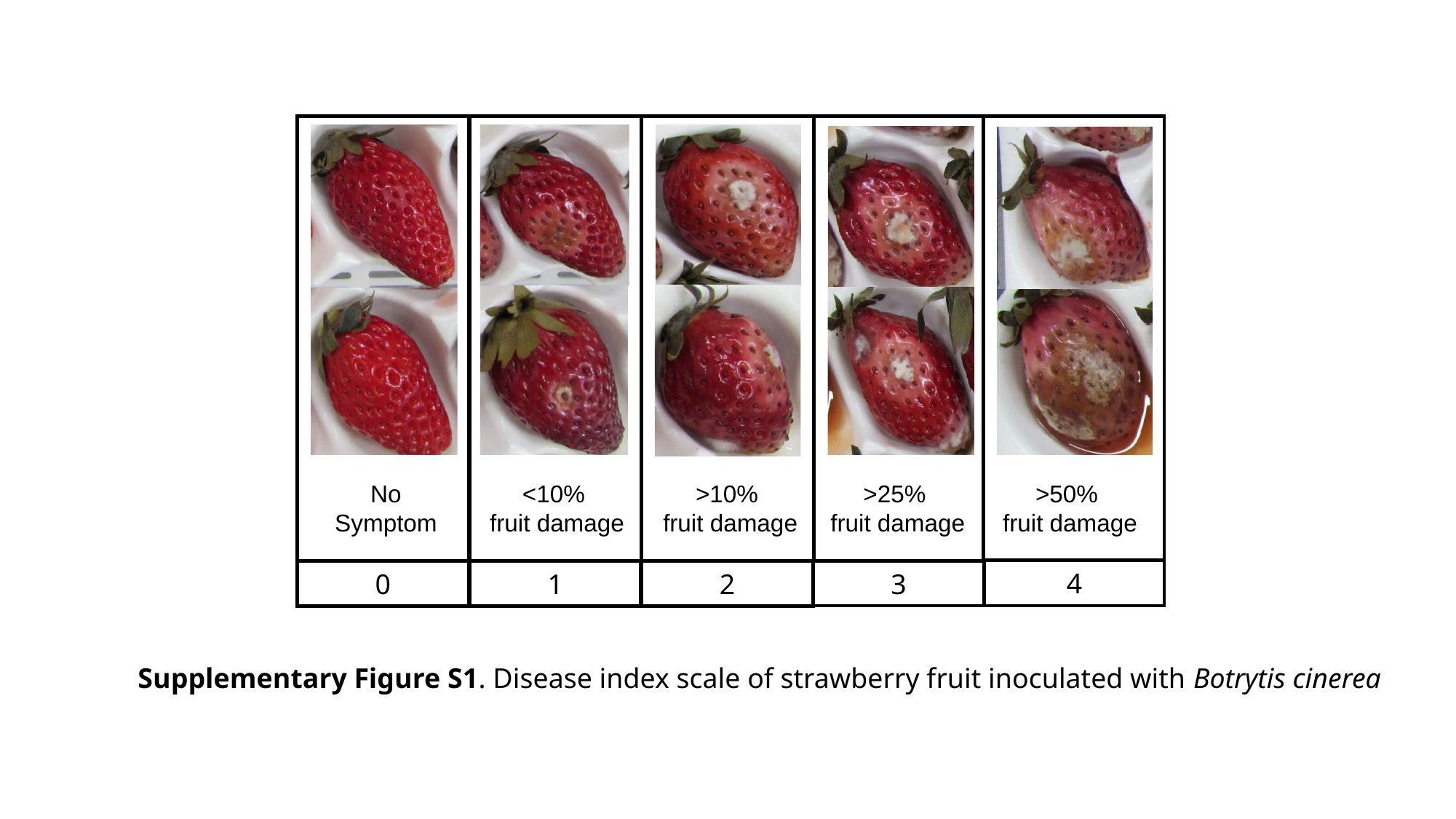

>50%
fruit damage
>25%
fruit damage
>10%
fruit damage
<10%
fruit damage
No
Symptom
4
3
1
2
0
Supplementary Figure S1. Disease index scale of strawberry fruit inoculated with Botrytis cinerea
